# Reimagining Hepatitis C Elimination: Time for Actions, Not Targets

**DOI:** 10.1002/jia2.70183

**Published:** 2026-07-28

**Authors:** Marina B. Klein, Nadine Kronfli, Jim Young

**Affiliations:** ^1^ Research Institute of the McGill University Health Centre Montreal Quebec Canada; ^2^ Department of Epidemiology Biostatistics and Occupational Health McGill University Montreal Quebec Canada

1

Global progress towards the World Health Organization's (WHO) 2030 hepatitis C virus (HCV) elimination targets has stalled. Only 13 countries are on track to achieve either relative (80% reduction in new acquisitions, 65% in deaths) or absolute (<5 new acquisitions/100,000, <2 deaths/100,000) targets, with only two of the 20 highest‐burden countries among them (China and Egypt) [1]. In 2024, 0.9 million people newly acquired HCV, a decline of only 8% from 2015; including nearly 10% of people who inject drugs (PWID) [2]. That year, the number of new acquisitions was about the same as the number cured [1]. Deaths from HCV‐related cirrhosis and cancer remained high at 240,000, a reduction of a disappointing 12% from 2015 [2].

HCV elimination is contingent on political will and adequate financing of national elimination programmes [1], with countries such as Spain, Australia and Egypt leading the way. Progress has been seen in low‐ and middle‐income countries (LMICs) such as Rwanda, Myanmar and Pakistan, but high‐burden countries such as Russia and the United States lag behind.

At this stage of the epidemic, continuing to do the same will not be enough. It is unrealistic to expect that countries which have endorsed targets but have failed to commit to or finance a national strategy will suddenly change course. We advocate for advancing specific concrete actions focused on key remaining challenges along the care cascade.

Step 1: Prevention. Transmissions associated with injection drug use continue to drive incident HCV. PWID are disproportionately concentrated in prisons and injection‐related risk‐taking increases following incarceration, due primarily to the scarcity of sterile injecting material. Expansion of opioid agonist therapy (OAT) and prison needle and syringe programmes (PNSPs) is critical, with the potential to have broader population‐level impacts [3].

*Challenge*: Many factors have undermined adoption and sustainability of OAT and PNSPs, including political and ideological opposition, security and confidentiality concerns, stigma, and chronic underfunding [4]. Even where available, these services are accessed by few, hampering their effectiveness [4].

*Action*: Where neither exists, OAT should be prioritized given its greater impact on reducing HCV transmission [3]. However, with a global shift in drug use towards non‐opiates [5], PNSPs will become increasingly crucial.

*Benefits*: By addressing multiple, interacting drivers of harm simultaneously, OAT and PNSPs interrupt the syndemic of substance use, infectious diseases, overdose and structural disadvantages that perpetuate poor health among incarcerated PWID, with health benefits extending beyond prison walls.

Step 2: Testing. As an increasing proportion of people who have been diagnosed are treated, the final stage of HCV elimination will require identifying those who remain undiagnosed, estimated to be 64% globally [2]. This fraction varies widely by country and risk population, and large data gaps persist, as few countries have comprehensive surveillance systems.

*Challenge*: As the number of those undiagnosed shrinks, the effort and cost of finding each additional case increases.

*Action*: Invest strategically in one‐time, test‐and‐treat campaigns, tailored to regional epidemiology, using point‐of‐care antibody screening combined with reflex RNA/antigen testing [6] or direct point‐of‐care RNA testing in high‐risk populations [7]. Countries with data‐driven testing strategies (e.g. Egypt, Rwanda, Iceland, Australia) have shown how to match HCV testing to the prevalence of undiagnosed acquisitions. Such campaigns are effective in diverse settings [8, 9].

*Benefits*: Testing campaigns provide faster identification, at lower cost, of those undiagnosed who can then be immediately offered treatment. Paired with hepatitis B testing [8], such campaigns also advance hepatitis B elimination.

Step 3: Treatment. Treatment benefits the individual, preventing liver disease progression and mortality. In the first comparative efficacy trial of pan‐genotypic HCV regimens, sofosbuvir plus daclatasvir was shown to be non‐inferior to sofosbuvir plus velpatasvir [10].

*Challenge*: The multiple sofosbuvir‐related patents, some extending beyond 2030 [11], are key obstacles to affordable pan‐genotypic regimens.

*Action*: With the expiry of the primary patent in 2024, advocacy for compulsory licensing of sofosbuvir might succeed in some countries. The Malaysian government issued a compulsory license for sofosbuvir in 2017, where it is being combined with ravidasvir [12]. But the risk of sanctions from drug companies and from the United States is a powerful disincentive [13]. In LMICs, advocacy may be best directed at getting governments to use existing agreements. For example, the Clinton Health Access Initiative and the Hepatitis Fund have an agreement with two generic drug manufacturers to supply 12 weeks of sofosbuvir/daclatasvir for $60 USD in 105 LMICs [14]. However, manufacturers may be reconsidering the agreement because it has been unexpectedly underused. In high‐income countries, treating just the wealthy while transmission continues among the poor, makes elimination impossible [13]. Payer license agreements [15] constrain drug costs, freeing up money for prevention and testing.

*Benefits*: Widespread uptake of curative and affordable treatments benefits not only the individual but the community via a treatment‐as‐prevention effect [16].

Step 4. Follow‐up. Mathematical modelling shows that as we approach elimination, an increasing percentage of new infections will be reinfections [17]. The high efficacy of treatment [10, 12] means resources should now be shifted from assessing cure to monitoring for reinfection to facilitate rapid retreatment.

*Challenge*: Guidelines typically recommend annual post‐cure HCV testing only in individuals with ongoing risk exposure. However, evidence suggests that reinfection rates are highest soon after successful treatment and transmission is likely to be clustered, because of high‐risk behaviours in relatively few individuals.

*Action*: Increased funding is needed for HCV RNA retesting after treatment. More nuanced guidelines should broaden definitions of ongoing risk. People at higher risk should receive at least biannual testing in the first few years after treatment [17, 18].

*Benefits*: As we approach elimination, identification and rapid retreatment of re‐infections will reduce the transmission that sustains the epidemic.

The aspirational HCV elimination targets have spurred many countries to implement actions that are having impact, but it is clear that targets will not be met globally by 2030. It is a bitter irony that, because HCV is curable, the thinking that HCV is “done” prevails at the very moment when renewed commitment is needed. Compounded by de‐investment in public health funding globally, not only will we miss targets but we risk undoing the progress that has been made.

Focused action, however, will yield significant benefits for individuals and populations over the coming years. Long‐term strategic investment is needed to ensure scale‐up of effective and sustainable interventions. Innovations such as a long‐acting HCV treatment (“one shot and done”) would be a potential game‐changer—if it can be made affordable. But the only time an infection has been eliminated is with a vaccine. Given pharmaceutical companies are retreating from HCV, innovation will require renewed public sector investment. It will take unrelenting advocacy from civil society partnered with the research and clinical communities to force governments to act.

The evidence‐based actions proposed above are by no means exhaustive, but the benefits are clear. Investment in elimination strategies has impact even beyond HCV, preventing drug‐related harms, addressing other sexually transmitted and blood‐borne infections, reducing health costs and improving the lives of millions of people worldwide.

## Author Contributions

MBK, NK, abd JY contributed ideas, drafted and reviewed the manuscript, and approved the final article.

## Conflicts of Interest

MBK has received funding for grants paid to her insitution from Abbvie Inc, ViiV Healthcare and Gilead Sciences. NK and JY declare no conflicts of interest.

## Data Availability

Data sharing not applicable to this article as no datasets were generated or analysed during the current study.
